# Factors Influencing the Quality and Extent of the Informed Consent Process in Patients Undergoing Surgical Procedures: A Cross-Sectional Study

**DOI:** 10.7759/cureus.68149

**Published:** 2024-08-29

**Authors:** Ruqaiya Al-Jardani, Wafa Al-Hadrami, Majda H Al-Balushi, Mudhar Al-Adawi, Samir Al-Nasseri

**Affiliations:** 1 Nursing, The Royal Hospital, Muscat, OMN; 2 Adult Critical Care Nursing Program, Higher Institute of Health Specialties, Muscat, OMN

**Keywords:** communication, patient satisfaction, decision-making, surgical procedures, informed consent, healthcare policies

## Abstract

Background

Informed consent is an essential component of medical practice, particularly in surgical procedures, as it guarantees that patients are thoroughly informed of the risks, benefits, and alternatives prior to receiving treatment. Nevertheless, research has demonstrated that patients frequently fail to recall critical information that was conveyed during the informed consent process, which emphasizes the potential for communication and comprehension deficiencies.

Objective

The objective of this study was to evaluate the quality of informed consent and the decision-making preferences of patients who are undergoing surgical procedures.

Methodology

A cross-sectional survey was conducted among 239 hospitalized patients who were scheduled for elective surgeries at a tertiary hospital in Muscat. The survey assessed patients' satisfaction with the process, preferences for decision-making, and comprehension of informed consent. Non-parametric correlation analysis was implemented to analyze the data.

Results

The consent form was signed by the majority of patients themselves (96.7%), with a median age range of 36-45 years and a relatively balanced gender distribution. For the remaining cases, where patients were unable to sign, the consent forms were signed by surrogates. The majority of patients were Omani nationals (96.7%), with a mixture of school and postgraduate education. The study did not identify any substantial disparities in the provision of informed consent between patients who received consent details in the outpatient clinic and those who received them in the ward. Although a small number of patients, particularly those with higher education, found the explanations unclear, the quality of the explanations was generally rated as explicit and sufficient. The ability to ask further questions, the degree of explanation, the explanation of alternative options, involvement in decision-making, the time to consider before making a decision, and the ability to repeat the explanation given were all positively correlated with satisfaction with the informed consent process.

Conclusion

The study emphasizes the significance of patient engagement and effective communication in the informed consent process. In order to improve patient satisfaction and comprehension, healthcare providers should prioritize interactive and comprehensive communication. It is imperative that policymakers establish policies that guarantee patients are afforded sufficient time and opportunity to comprehend the risks, advantages, and alternatives of surgical procedures.

## Introduction

Internationally, informed consent encounters numerous obstacles [1]. The challenges include a lack of training and a sense of responsibility regarding the criticality of the consent procedure, which is causing surgeons and pre-operative nurses to not take the consent aspect seriously [2]. The literature also indicates that the patient's comprehension of the consent procedure may be influenced by their level of education [3]. Schenker et al. discovered that patients were not adequately informed prior to entering the operating room, which resulted in them asking numerous inquiries [4]. In another study, it was discovered that surgeons were unable to provide patients with adequate information due to time constraints, which exacerbated the informed consent process [5]. The absence of an explanation of the adverse effects of surgery was the cause of 30.4% of the reported consent failures, according to a study conducted in the United States [6]. In the same vein, Loughran et al. observed that surgeons frequently neglected to furnish their patients with comprehensive information regarding the advantages and disadvantages of the associated surgery, despite the existence of explicit informed consent policies, in the United Kingdom (UK) hospitals [7].

Additionally, the patient and their family are occasionally not informed of the dangers and complications associated with surgery [8]. Delays that result from incomplete or inaccurate consent forms can contribute to patient dissatisfaction and an increase in the surgery waiting list, thereby increasing hospitalization time and costs [7]. Furthermore, patient safety is jeopardized by the potential for patient disability or mortality as a result of incomplete or inaccurate consent forms [7].

The consent form in the Ministry of Health, Sultanate of Oman, is a source of increased concern due to the fact that the quality of the consent is not always met. Quality of consent refers to the extent to which informed consent procedures ensure that patients fully understand the risks, benefits, and alternatives of a surgical procedure. Quality consent is met when patients are provided with comprehensive, accurate, and clear information, allowing them to make well-informed decisions about their care. This involves a thorough discussion between healthcare providers and patients, covering potential risks, complications, and outcomes of the surgery.

The importance of quality in consent cannot be overstated, as it supports patient autonomy and trust in the healthcare system. Without quality consent, patients might face unexpected complications, which can lead to dissatisfaction, increased hospitalization times, and higher medical costs. Inadequacies in consent processes, as observed in the Ministry of Health, Sultanate of Oman, can result in patients and their families not being fully informed, thus risking patient safety and well-being The quality of the informed consent procedure in the territory hospital is being called into question as patients are receiving inadequate details and accurate information. Consequently, the objective of this investigation was to evaluate the quality of informed consent and the decision-making preferences of patients who are undergoing elective surgical procedures.

## Materials and methods

A cross-sectional survey was conducted to collect data from patients who were undergoing elective surgical procedures in specific departments of a tertiary hospital in Muscat. Surveyors were present to clarify any ambiguous inquiries. Prior to moving on, a comprehensive pre-testing was conducted on the initial 10 patients to verify their comprehension. The question design was improved through the use of face validity criteria to ensure that all ambiguity was completely avoided.

The survey centered on the patient's capacity to recall details regarding risks and alternative treatment options, their preferences towards decision-making processes, and their general satisfaction with the informed consent procedure. Further inquiries were conducted on demographic information, educational background, the type and date of the procedure, the urgency of treatment, and the need for translators. Patients were interviewed in various hospital departments prior to the surgery, typically within one or two days of giving their informed consent. The subjective comments provided by patients were documented on the back of the survey form. The survey was completed within around 15 minutes.

Setting

The study was conducted in the surgical wards of a tertiary hospital in Muscat. This facility provides services for 630 in-patients who are admitted through different departments, such as child health, medicine, surgery, obstetrics and gynecology, and oncology. The institution has expertise in performing complex surgical procedures.

Study participants

The target population comprised preoperative patients in the general surgery, urology, and obstetrics and gynecology departments. The eligible participants consisted of persons between the ages of 18 and 60 who possessed the mental ability to sign consent forms and were literate in either Arabic or English. The sampling process employed probability sampling techniques. The sample was stratified based on surgical specialization, and a simple random sampling method was employed to estimate the sample size within each stratum. Proportionate stratified sampling was employed to ensure homogeneity. The sampling frame was derived from the healthcare information management system named Al-Shifa System, utilizing specific criteria for inclusion and exclusion. The inclusion criteria consisted of patients who were scheduled for elective procedures and provided their consent to participate. Conversely, the exclusion criteria covered patients from other departments, emergency surgeries, children under the age of 18, and those who required additional surgery because of difficulties. The sample size calculation employed the Z alpha formula [9], yielding a minimum sample size of 384. In order to compensate for a 10% attrition rate and enhance statistical power, the final sample size was augmented to include 422 patients.

Procedures for recruitment and participation

After receiving ethical approval from the hospital's research committee, the researcher requested consent from the unit managers of the surgical departments. Recruitment and data collection were conducted concurrently as a result of the limited duration of the preoperative admission stay. Prior to data collection, patients were given informed consent. The researcher and four nurse assistants from each surgery department conducted the interviews.

Data collection

Self-report questionnaires were used to collect data before the surgery in order to evaluate the quality of informed consent and the decision-making preferences of patients who are undergoing elective surgical procedures. The study was carried out in surgical wards and surgical outpatient departments (OPD), with assistants who were instructed on the objectives of the research, the processes for collecting data, and the need to keep anonymity.

The data-gathering tool utilized was a structured questionnaire adapted from Brezis et al. [10]. It consisted of two sections. Section A consisted of demographic information, including gender, type of surgery, level of education, occupation, and employment status. Section B included inquiries pertaining to patient comprehension of informed consent, details offered to the patient, permission for the procedure, satisfaction with the decision-making process, participation in decision-making, awareness of rights, and sufficiency of time for explanations.

Validity and reliability of the questionnaire

A pilot study was conducted with 10 patients to evaluate the validity and reliability of the questionnaire, ensuring its suitability for the main study. Modifications were implemented and modifications were made to ensure the accuracy and relevance of the content, taking into account the comments received during the pilot research.

Data analysis

The data underwent cleaning, coding, and entry into Statistical Product and Service Solutions (SPSS, version 22.0; IBM SPSS Statistics for Windows, Armonk, NY) for the purpose of statistical analysis. The data were summarized and described using descriptive statistics, specifically frequencies and percentages. The results were then presented in tables.

Ethical considerations

The study aims to enhance the quality and clarity of information provided to patients, leading to several benefits for participants. Increased awareness allows participants to gain a better understanding of the risks, benefits, and alternatives associated with their surgical procedures, resulting in more informed decision-making. Improved safety is achieved by ensuring that consent forms are complete and accurate, reducing the likelihood of complications and misunderstandings during and after surgery. Enhanced trust between patients and healthcare providers is fostered through better communication and transparency, improving the overall patient experience. Additionally, knowing that they are fully informed can help reduce the anxiety and stress associated with undergoing surgery. The hospital's ethical committee granted ethical permission. Prior to obtaining consent, participants were briefed on the purpose, characteristics, and protocols of the study. The study ensured privacy and confidentiality by coding and securely storing all data to protect anonymity.

## Results

Over a three-month period, a total of 470 patients were registered for elective surgery, but only 239 patients appeared for their procedures. The majority of participants said that they signed the consent form by themselves, with 96.7% (standard deviation = 0.18) reporting this and the remaining cases by their surrogates, as presented in Table 1. Patients’ median age ranged from 36-45 years (standard deviation = 0.96) (Table 1).

**Table 1:** Demographic characteristics of the participants

Characteristics	N (%)	Median	Standard deviation
Age		36-45 years	0.96
18-25	22 (9.2%)		
26-35	74 (31%)		
36-45	73 (30.5%)		
46-60	69 (28.9%)		
Gender		Female	0.49
Male	107 (44.8%)		
Female	132 (55.25)		
Nationality		Omani	0.18
Omani	231 (96.7%)		
Non-Omani	8 (3.35)		
Education level		Higher Education	1.6
Below school education	29 (12.25)		
School education	105 (43.9%)		
Higher education	105 (43.9%)		
Informed consent signature		Yes	0.18
Yes	231 (96.75)		
No	8 (3.35%)		
Satisfaction level		Satisfied	0.8
Highly satisfied	78 (32.65%)		
Somewhat satisfied	43 (185%)		
Satisfied	109 (45%)		
Not satisfied	9 (3.8%)		

Descriptive statistics were done to analyze the patients’ demographic characteristics and the place where informed consent was taken. There were no statistical differences among patients who received the consent details in the OPD when compared to those given in the ward (Table 2).

**Table 2:** Demographic details for personnel providing informed consent to either the outpatient clinic or in the ward before surgeries *OPD = Outpatient Department. A p-value <0.05 was considered statistically significant.

Characteristics	Personnel providing the informed consent details		P-value
	Surgeon/Nurse in OPD	Physician/Nurse in the ward	
Age			0.06
18-25	14	8	
26-35	55	19	
36-45	53	19	
46-60	45	23	
Gender			
Male	80	30	0.6
Female	87	43	
Education			0.29
Uneducated	22	7	
School education	77	27	
University education	68	36	

Table 3 shows the level of explanation and clarity of the informed consent among patients. Interestingly, the majority of patients reported that the explanation was clear enough and very few of them reported a poor explanation, especially those who had higher education (five patients). However, none of these were statistically significant between the groups.

**Table 3:** Contingency table for the demographic characteristics with the degree of explanation *A p-value <0.05 was considered statistically significant.

Variable	To what degree the explanation you received was clear, partially clear, or not clear?			P-value
	The explanation was clear	The explanation was partially clear	The explanation was not clear	
Level of education				0.86
Uneducated	23	5	1	
School education	84	18	1	
Higher education	80	20	5	
Age				0.73
18-25	16	5	0	
26-35	62	11	1	
36-45	54	15	3	
46-60	54	12	3	
Gender				0.1
Male	79	25	2	
Female	108	180	5	

A nonparametric correlation analysis was conducted to investigate the relationship between the level of satisfaction with the informed consent and other variables, using a two-tailed significance test. A statistically significant moderate positive connection (p = 0.001) was seen between satisfaction and the ability to ask more questions following the explanation of the surgical procedure. Despite the weak positive association discovered between satisfaction level and gender, degree of involvement, and ability to repeat knowledge, these relationships were statistically significant, as indicated in Table 4.

**Table 4:** Correlation between satisfaction from the informed consent and other variables *A p-value <0.05 was considered statistically significant.

Variable	How satisfied you are with the quality of the informed consent given to you?	P-value
Gender	0.23	0.001
Degree of explanation (clear, sufficient, detailed)	0.19	0.003
Explanation of alternative options available	0.216	0.001
Degree of involvement in the decision	0.285	0.001
Time to think before taking a decision	0.26	0.001
Ability to repeat explanation given	0.226	0.001
Ability to ask questions after an explanation	0.379	0.001

## Discussion

The consent form was signed by the majority of the patients for elective surgery in the study (96.7%), and the rest were signed up by their surrogates due to difficulty in writing. Participants' median ages ranged from 36 to 45 years. The gender distribution was relatively balanced, with 55.2% of participants being female and 44.8% being male. The majority of patients were Omani nationals (96.7%), and their educational backgrounds were evenly distributed between school and postgraduate education (43.9%). Patients who received consent details in the OPD and those who received them in the ward did not exhibit any substantial disparities in the provision of informed consent.

The findings suggest that, although most patients reported receiving comprehensive and satisfactory explanations during the informed consent procedure, a subgroup of patients, notably those with higher levels of education, perceived the explanations as inadequate. Patient satisfaction was much improved by the capacity to inquire and obtain comprehensive explanations. The results emphasize the significance of clear communication and active involvement of patients in the process of obtaining informed consent. This is crucial for enhancing overall satisfaction and ensuring that patients have a comprehensive understanding before undertaking surgical treatments. Research indicates that patient knowledge and understanding of surgery are important components of satisfaction with the decision to proceed with surgery [11].

With respect to the quality of the explanations, the majority of patients reported that they were straightforward and adequate. A small number of individuals, particularly those with higher education, expressed that the explanations were unclear. Studies show a significant correlation between the opportunity to ask questions and satisfaction with informed consent [12]. A moderately positive correlation between the potential to ask further questions and satisfaction with the informed consent was identified through non-parametric correlation analysis, which was highly significant (p = 0.001). Satisfaction was also significantly correlated with other factors, including the degree of explanation, explanation of alternative options, involvement in decision-making, time to consider before making a decision, and the ability to repeat the explanation given [13].

Our investigation evaluated the quality of informed consent and the decision-making preferences of patients who were undergoing surgical procedures. The findings suggest that a significant number of patients signed the consent form; however, a significant number of them did not recall receiving comprehensive explanations about the risks and alternatives associated with their procedures. This discovery is in accordance with prior research that has demonstrated that recall from the informed consent process is frequently inadequate as a result of the challenges associated with understanding the information that is provided [10].

In our survey, 96.7% of patients reported signing the informed consent form; however, a substantial number of patients did not recall receiving comprehensive information regarding alternatives or complications. This matter is of the utmost importance, as the informed consent process is intended to guarantee that patients comprehend the procedure and consent voluntarily. Our results are consistent with those of Brezis et al. who also discovered that half of the patients did not recall receiving explanations about risks, and two-thirds did not recollect discussions of alternative options [10]. This suggests that the sheer acquisition of a signature is insufficient to achieve genuine informed consent.

Quality of informed consent and clinical setting

The demographic characteristics of patients receiving consent information in outpatient clinics were not significantly different from those in the ward, as demonstrated by our study. Nevertheless, it appeared that the clinical environment had an impact on the perception of risk and alternatives. Despite the absence of a comprehensive discussion of specific risks and alternatives, patients frequently perceived the explanations as explicit and detailed. This implies that patients may prioritize other aspects of the treatment, such as the technical intricacies of the procedure, anesthesia, or recovery, over the inherent risks. For instance, a study evaluating the quality of informed consent for invasive procedures found that many patients did not recall receiving explanations about risks or alternative options, indicating that the perception of being well-informed may not always correlate with actual comprehensive discussions [10].

Additionally, research into risk perception and risk attitude in informed consent processes showed that decision-makers' perceptions of risk can vary greatly, often influenced by how information is framed, further suggesting that the clinical environment and communication style significantly affect patient perceptions [14]. Moreover, an analysis of patient perceptions in a high-context communication culture revealed that, while extensive information on benefits and post-procedure issues was desired, risks and alternatives were often perceived as less critical, highlighting a potential discrepancy between patient expectations and actual practice [15]. Finally, in a cross-sectional survey on the quality of informed consent in cancer clinical trials, it was found that, while most patients considered themselves well-informed, many did not recognize key risks and uncertainties associated with trial participation, emphasizing the importance of thorough communication [16].

The clinical environment significantly impacts patients' perceptions of informed consent, often leading them to prioritize the technical details of the treatment over the inherent risks. This underlines the need for tailored communication strategies to ensure a comprehensive understanding of all aspects of treatment and consent.

Satisfaction with the informed consent process

The informed consent process was generally well-received, even though there were rejects in the recall of specific details. This inconsistency may be attributed to the patients' perception of fluency and the sufficiency of the explanations provided for certain aspects of their treatment, despite the fact that critical elements such as risks and alternatives were disregarded. The high satisfaction levels may mask specific deficiencies in the quality of treatment, a phenomenon that has also been observed in previous research.

Perceived objective of informed consent

Understanding patients' perceptions of the objective of informed consent is essential for improving its practice. Further insight into the perceptions of informed consent discovered that patients' perspectives regarding the purpose of informed consent are diverse, with the predominant perspective being the facilitation of patients' self-decision-making [17]. The survey of 488 adults conducted by the researchers revealed that the most reflective purposes of informed consent were "help patient decide" and "make sure patient understand" that patients rated. This is consistent with our discoveries that patients prioritize comprehensive and transparent information to make well-informed decisions. Nevertheless, the disparity between patients' perceptions and the actual practice of informed consent underlines a need for improvement.

Correlations and statistical significance

Significant factors that affect patient satisfaction were identified by our non-parametric correlation analysis. The capacity to ask questions after the explanation was found to have a moderate positive correlation with satisfaction, which was highly significant (p = 0.001). The ability to repeat information, gender, and the degree of involvement were also significant correlations (p = 0.001). The significance of patient engagement and straightforward communication in the decision-making process is emphasized by these findings. These results are consistent with prior research that has highlighted the importance of patient involvement in decision-making and explicit adaptable communication strategies in order to improve satisfaction [18,19]. Patient-centered care practices are reinforced by the positive correlation between satisfaction and the capacity to pose questions. Conversely, Brezis et al. discovered deficiencies in the quality of informed consent, as numerous patients were unable to recall discussions of alternative options or explanations of their risks [10]. This emphasizes the necessity of enhanced communication strategies that are customized to the unique preferences of each patient. It has been highlighted that the inadequacy of written informed consent alone, positing that oral explanations are more effective in ensuring patient awareness [12]. Alsaffar et al. discovered that patient recall of perioperative risks was not substantially enhanced by procedure-specific handouts, emphasizing the necessity of alternative educational strategies [20].

Moreover, a systematic review was conducted to examine the efficacy of health literacy interventions in the informed consent process, which revealed that patients' comprehension, information retention, and satisfaction with the consent process may be improved through health literacy interventions, including the incorporation of multimedia formats and the enhancement of the intelligibility of consent materials [21]. The research determined that customized health literacy interventions are essential for guaranteeing that patients comprehend the information they are given and can make well-informed decisions regarding their care.

A study in South Africa demonstrated that, despite the fact that patients were generally cognizant of their right to informed consent, a significant number of them encountered obstacles, including poverty, language barriers, and low educational levels [22]. The majority of patients expressed a preference for comprehensive disclosure of all material hazards and a desire for healthcare professionals to possess improved communication skills. The significance of enhancing communication and resolving socioeconomic factors to facilitate informed or shared decision-making in healthcare settings was emphasized by the study.

Study strengths and limitations

Despite the constraints associated with this investigation, several strengths merit attention. First, the study’s high response rate and the balanced gender distribution among participants provide a representative sample of the patient population in a tertiary hospital setting. Second, the inclusion of both outpatient and ward settings enhances the applicability of the findings across different clinical environments within the hospital. Third, the use of non-parametric correlation analysis adds robustness to the identification of factors that influence patient satisfaction with the informed consent process. These strengths contribute to the study’s valuable insights into the quality of informed consent and patient decision-making preferences.

There are numerous constraints associated with this investigation. The generalizability of the findings to other contexts may be impacted by the fact that the sample size was restricted to a single tertiary hospital in Muscat, despite being sufficient for the analyses conducted. In addition, the study utilized self-reported data from patients, which may be susceptible to social desirability bias or recall bias. The cross-sectional design of the study also restricts the capacity to infer causal relationships from the observed correlations.

Implications for clinical practice and policy

The findings of this investigation have substantial implications for policy and clinical practice. Healthcare providers should prioritize interactive and comprehensive communication during the informed consent process, as evidenced by the high satisfaction levels reported by patients who had the opportunity to pose questions and receive detailed explanations. Policies should be established to guarantee that patients are afforded sufficient time and opportunity to comprehend the dangers, advantages, and alternatives of surgical procedures. The quality of informed consent should be improved by emphasizing the significance of effective communication and patient engagement in healthcare provider training programs.

## Conclusions

In order to improve the generalizability of the results, future research should investigate larger, multicentre studies. Longitudinal studies could offer more profound insights into the causal relationships between patient satisfaction with informed consent and communication practices. Furthermore, qualitative research that investigates deeper into the experiences and preferences of patients could provide valuable insights for customizing informed consent processes to accommodate a wide range of patient requirements. Additional research into the specific obstacles encountered by patients with higher education in comprehending medical explanations could facilitate the development of targeted interventions that enhance the clarity and efficacy of informed consent communication.

The study stresses the importance of patient engagement and effective communication in the informed consent process. The satisfaction and comprehension of patients can be substantially improved by providing them with the opportunity to ask questions and receive detailed explanations. In order to enhance the quality of informed consent and, as a result, patient outcomes, healthcare providers and policymakers should prioritize the implementation of strategies that encourage interactive and patient-centered communication.
